# Improving health equity: changes in self-assessed health across income groups in China

**DOI:** 10.1186/s12939-018-0808-y

**Published:** 2018-07-03

**Authors:** Yuqi Zhou, Xi Yao, Weiyan Jian

**Affiliations:** Department of Health Policy and Management, Peking University School of Public Health, Xueyuan Road 38, Haidian District, Beijing, 100191 China

**Keywords:** Health equity, Self-assessed health, Concentration index, Income group, China

## Abstract

**Background:**

Beginning in 2010, China has endeavoured to expand health coverage and provide residents with fair access to primary health care with the intention of improving health equity. This study aims to measure changes in income-related health inequity in China between 2010 and 2014.

**Methods:**

Data were extracted from the nationally representative annual survey of the China Family Panel Studies in 2010 and 2014 with a first wave of 31,743 respondents and a second wave of 32,006 respondents over age 15. In both years, subjects were stratified into the following five categories of income: poorest 20%, lower 20%, medium 20%, higher 20% and richest 20%. The concentration curve and index was used to compare the distribution of health status in income quintiles, and a logistic model was used to examine the relationship between health and socioeconomic indicators with self-assessed health as the primary outcome of interest.

**Results:**

Income was significantly associated with self-assessed health in China. The concentration curve was above the line of equality in both years, while the self-assessed health line in 2014 was closer to the equality line. The concentration index (CIN) displayed the similar result of decreasing inequality, with the CIN in 2014 (− 0.157) closer to zero (the line of equality) than that of 2010 (− 0.167). In 2010, there was a decreasing trend of people reporting poor health from the poorest to the richest, while in 2014, there was no significant difference between the poorest and lower 20% or between the higher 20% and the medium 20%. The odds ratio of the prevalence of self-reporting poor health between the poorest and richest increased from 0.555 (95% CI: 0.484–0.636) in 2010 to 0.598 (95% CI: 0.513–0.696) in 2014.

**Conclusions:**

From 2010 to 2014, the self-assessed health gap between income groups in China decreased, and health equity improved. However, health differences remain. In order to achieve better health for all, China should further strengthen the role of primary care in reducing health inequity.

## Background

Health equity emerged as a global public health priority following the Alma-Ata Primary Health Care Summit in 1979 when agreement was reached on a strategy for achieving the goal of health for all. Equity in health means that everyone should have a fair opportunity to attain his or her full health potential [1]. This opportunity requires the absence of systematic disparities in health or in its major social determinants between groups with different levels of underlying social advantage or disadvantage—that is, wealth, power, or prestige [2]. The World Health Organization (WHO) defines health as a physical, psychological and social state of integrity. As a basic human right, it does not vary according to gender, race, politics, faith, social-economic class or life and working conditions [2].

However, inequalities in health among different countries, regions and populations remain prevalent [3]. The WHO has set the goal of closing the gap in a generation, and there is a long way to go to improve health equity [4]. China has made great effort in increasing the total amount of medical and health services resources [5]. For instance, number of medical technicians and staff per thousand people rose from 3.63 people in 2000 to 5.40 people in 2014, and the number of beds per thousand in medical and health institutions increased from 2.38 in 2000 to 4.77 in 2014. With these inputs, China has witnessed remarkable achievement in prolonging the life expectancy nearly five years since 2000, declining by two thirds the under-five mortality rate and reducing by three quarters the maternal mortality ratio from 1990 to 2015, and endeavouring to control and treatment of epidemic diseases such as HIV/AIDS, malaria and tuberculosis, which meets the Millennium Development Goal and is highly praised by the international counterparts [5]. However, complaints about unaffordable health services due to high out-of-pocket health expenditures have gradually increased. Health inequity is appearing as the priority of health issues and attracts extensive attention. China Health and Nutrition Survey found that average Height-for-age z-scores (HAZs) for Children has improved from 2000 to 2009, but inequality widened [6]. National Survey of the Aged Population in Urban/Rural China pointed out there was inequitable health care utilization and health outcomes for the elderly in self-assessed health status, physical functions and psychological wellbeing from 2006 to 2009, which was generated by fragmented health insurance schemes [7]. Moreover, there are inequalities of utilization for maternal health services in western rural area and rural migrant workers [8, 9]. Encountered with health problems in unbalanced distribution of health resources, great health status variances among different regions and different populations, and unfair utilization of health services, the government has adopted a series of measures on narrowing the rich-poor and rural-urban gap to improve health equity.

In April 2009, Chinese central government launched the plan to deepen the reform of the medical and health care system with the goals of providing universal coverage of essential health services for all citizens by 2020 [10]. Substantial initiatives have been implemented in public hospital reform, expansion of health insurance and strengthening of primary care. Specifically, the following three measures are oriented to improve health equity [11]:

First, equalize essential public health services. China has established a basic public health service package, including 21 items of services in 9 categories, which covers residents health records, health education, vaccination, infectious disease prevention, child care, maternal health care, elderly health care, management of chronic diseases and severe mental illnesses. Public financing allows all Chinese people to receive these services free of charge, with per capita subsidy increased from CN¥ 15 (US$ 2.4) in 2010 to CN¥ 35 (US$ 5.5) in 2014 [12].

Second, strengthen capacity in primary care institutions, which are the main providers of basic public health services. Between 2009 and 2011, CN¥ 60 billion (US$ 9.4 billion) was allocated to establish over 33,000 new regional health care clinics, mostly in rural and western China [13]. Essential drug system was built to improve basic drug reserves and standardize its medication. Professional team of general practitioners and nurses was trained to promote primary care delivery. Strengthening their capabilities will help to ensure the accessibility and quality of primary health services.

Third, further expand health insurance coverage through public financial subsidies. According to the 2015 National Health Statistic Yearbook [13], from 2010 to 2014, health insurance coverage increased from 94.6 to 97.5% and the reimbursement rate fluctuated around 70%. The subsidy per capita for Urban Residents Medical Insurance (URMI) and New Rural Cooperative Medical System (NRCMS) rose from CN¥ 120 (US$ 18.8) in 2010 to CN¥ 320 (US$ 50.2) in 2014.

All of these policies are expected to enhance the affordability and accessibility of health services for low-income people in underdeveloped areas and thus improve health equity. However, whether this has been achieved requires the examination of empirical evidence. To our best knowledge, there is limited evidence about the effect of these health reform measures on health equity. This study focuses on the changes in self-assessed health among different income groups in China from 2010 to 2014 and measures the health equity of the Chinese people during this period of intensive introduction of health equity policies to strengthen capacity of primary health care and reduce disease financial burden.

Self-assessed health (SAH) is one indicator of health status [14, 15]. In recent years, more studies have emerged with self-assessed health reported to be significantly associated with communicable diseases, non-communicable diseases, and mortality [16–19]. In particular, SAH is more sensitive than objective disease indicators when reflecting mental conditions such as pain, fatigue, and depression [20, 21]. And SAH also suggests potential illness and its development in the near future [22]. As with various biomarkers for a number of diseases or health conditions, self-assessed health is widely used as a measure of health [23, 24]. In China studies have shown that self-assessed health has high reliability and stability in measuring the health level, and it is suggested that self-assessed health is more sensitive for predicting health than the commonly used indicators of prevalence of acute illness in the past two weeks and chronic diseases, especially for the rural elderly in a large population [25–27]. Since SAH is reported based on a simple question “How would you rate your health status”, it is a more feasible and inclusive measure than guided physician examinations, which may capture subtle elements of health information that is difficult to assess by routine physician reports. SAH, the aggregated health measure, is frequently employed in health economic analysis.

## Methods

### Data and sample

China Family Panel Studies (CFPS) is a nationally representative, annual longitudinal survey of Chinese communities, families, and individuals launched in 2010 by the Institute of Social Science Survey (ISSS) of Peking University, China [28]. The baseline survey was conducted from April 2010 to September 2010 and the 2014 annual survey lasted from July to December 2014. CFPS employed multi-stage probability sampling with implicit stratification to create a nationally representative sample from 25 provinces. Five large provinces or municipalities (Guangdong, Gansu, Liaoning, Henan, and Shanghai) were chosen for initial oversampling and the remaining 20 provinces or municipalities were grouped together so that regional comparisons could be made. County, village and household subsamples were drawn with probability-proportional to size sampling. In the first stage, 162 counties or equivalents were sampled. Then, 649 administrative villages were sampled. Ultimately, 19,986 households were sampled from the chosen villages. The sample weights were the inverse of multiplying the sampling probability at each stage. In 2010, the CFPS successfully interviewed 42,590 including 33,600 adults and 8990 children and had a response rate of 84.1%. In 2014, 37,147 adults and 8617 children were selected after careful weighting. In both years, trained interviewers collected data face-to-face using the questionnaire designed to collect individual-, family-, and community-level longitudinal data in contemporary China. The CFPS questionnaires contain a wealth of information covering economic activities, education outcomes, family dynamics and relationships, migration, and health. This research combined the databases of adults and families from the CFPS 2010 baseline survey and the 2014 annual study, yielding a sample of 31,743 and 32,006 respondents aged over 15, respectively. More than 30% of respondents in 2014 were newly enrolled after tracking back information in 2010 by matching the unique personal identification codes. Taking into consideration the high rate of withdraw and enrolment of new interviewers in 2014, it was treated as two cross-section studies rather than a longitudinal study in data analysis.

### Variables

This study aims to explore the differences in income-related health equity, with other demographic variables, socioeconomic status, and geographical distribution differences as control variables. Specifically, Self-assessed health. Considering the increasing importance of self-assessed health in predicting individual health status, this variable was the primary health outcome of interest. In the survey, participants were asked ‘how would you rate your health status?’ and it was measured on a standard 5-point scale categorized by healthy, fair, relatively unhealthy, unhealthy and very unhealthy [28]. Similar to prior studies [25–27], self-assessed health was dichotomized into good (healthy and fair) and poor (relatively unhealthy, unhealthy, and very unhealthy) health.

Income. Income quintile groups, commonly-used in social statistics, were the proxy representing subjects’ social-economic class [29]. Given that younger and older groups in China are usually financially challenged, especially students and retirees, average household income was used to reflect individuals’ financial status. Household income included working wages, assets, monetary gifts, pensions, and any other forms of subsidy. Due to the large economic development disparities among different provinces in China, it is difficult to judge the local economic level where the family position in terms of the absolute value of a household income. Therefore, we have changed income indicator from absolute value to “relative value”. Specifically, it was compared with average provincial income to generate a measure of relative household income, which was then divided into the following 5 quintiles: poorest 20%, lower 20%, medium 20%, higher 20% and richest 20%.

Other variables. Age, gender, marital status, education level, occupation, region (western, central or eastern China) and residence (urban/rural) were included as control variables, as a number of studies have explored their association with health [4, 8, 26, 30]. District and residence differences are also important when discussing health inequity in China. Education levels consisted of being illiterate, having completed junior high school or below, having completed vocational school and below, and having completed a bachelor degree and above. Occupational status was defined as unemployment, agricultural workers, self-employed workers and employees.

### Statistical analyses

Data were analysed using STATA software (version 13.0). This study used concentration curves, a concentration index and logistic regression to analyse health equity.

Concentration curves plot the cumulative percentage of self-assessed health (y-axis) against the cumulative percentage of the sample [31, 32] ranked by income level from the poorest to the richest (x-axis) in 2010 and 2014, and they provide a visual impression of income-rated inequality in the distribution of self-assessed health over time. If everyone, irrespective of his or her income level, has exactly the same health status, the concentration curve will be the line of equality, and it will run from the bottom left-hand corner to the top right-hand corner. When the concentration curve lies above the line of equality, the self-assessed health is higher among poorer people, and the farther the curve is above this line, the more concentrated the health values are among the poor.

The concentration index (CIN) is defined as twice the area between the concentration curve and the line of equality [31], with reference to the concentration curve. The concentration index can be conveniently formulated by

1 $$ \mathrm{C}=\frac{2}{\mu}\;\mathit{\operatorname{cov}}\left(h,r\right) $$

where h is the self-assessed health value, u is its mean, and r = i/N is the fractional rank of individual i in the distribution of income level, with *i* = 1 for the poorest and i = N for the richest. After controlling for the confounding effect of demographic characteristics, standardization of the concentration index is obtained from the regression:

2 $$ 2{\sigma}_r^2\left(\frac{h_i}{\mu}\right)=\alpha +\beta {r}_i+{\sum}_j{\delta}_j{x}_{ji}+{v}_i $$

where ${\sigma}_r^2$ is the variance of the fractional rank, *x*_*j*_ are the confounding variables, for example, age, sex, etc., and the OLS estimate $\widehat{\beta}$ is an estimate of the indirectly standardized concentration index. In addition to identifying socioeconomic inequality in health, the concentration index can give a measure of comparable magnitude of inequality across different groups, such as time periods and regions [31].

The logistic regression model was used to assess the association between income levels and the likelihood of self-reporting poor health after controlling for other demographic variables. The regression formula was

3 $$ Y={\alpha}_0+{\beta}_i{\sum}_{i=2}^5{Income}_i+\upgamma \sum {X}_j+\varepsilon $$

where *Y* is the dependent variables representing dichotomous self-assessed health of 1 for poor health (relatively unhealthy, unhealthy, and very unhealthy) and 0 for good health (healthy and fair); *Income*_2_ to *Income*_5_ represents the lower, medium, higher and richest income groups, respectively, with *Income*_1_ (poorest) as the reference; *X*_*j*_ is the control variable in the regression equation representing other demographic characteristics such as age, gender, marital status, occupation, education, residence (urban and rural) and region (western, central and eastern China); *ε* means residual error.

## Results

### Population characteristics

Tables 1 and 2 show the study population characteristics in 2010 and 2014. The sample included 31,743 respondents in 2010 and 32,006 in 2014, with a similar mean age of 45.5 (SD 16.39) and 45.3 (SD 17.42), respectively. However, the age structure differed significantly between the years (X^2^ = 307.23, *P* < 0.001). In comparison, people aged 50 to 60 (15.88%) and above age 60 (19.49%) made up a larger proportion of the population in 2014 than they did in 2010, while the proportion of people age 15 to 50 (67.62%) was higher in 2010. In both years, over half of respondents were men, though the proportion of female subjects was slightly greater in 2014 (49.53%) than in 2010. In 2014, more people lived in eastern China (48.52%). Western and central residents dropped from 27.35% of respondents in 2010 to 26.45% in 2014, and 25.51% in 2010 to 25.03% in 2014, respectively. Similarly, urban citizens outnumbered rural citizens in both years, and rural residents decreased from 49.75% in 2010 to 43.67% in 2014. In terms of education attainment, subjects with less than a junior high school education dominated in both years, with the rate of illiteracy and semi-literacy amounting to approximate25%. In contrast, 19.60% reported having a high school education or greater in 2014, which was slightly less than the 20.34% who reported this education level in 2010. Approximately 23% of respondents reported being single in 2010, and this rate rose to 27.17% in 2014. As for occupation, 49.96% were jobless in 2010, but only 26.39% were unemployed in 2014. Of those with jobs most people were employees in 2014, while more people were engaged in agriculture work in 2010. Approximately 7% of respondents were self-employed in 2010, compared to 10.83% in 2014. In terms of self-assessed health, there was improvement in the proportion of people self-reporting good health, with the increase from 85.44% in 2010 to 86% in 2014.

**Table 1:** Descriptive statistics stratified by year

Characteristic	Year 2010			Year 2014			X^2^	P*
	N(%)[SE]^a^ (*n* = 31,743)			N(%)[SE]^a^ (*n* = 32,006)				
Age							307.23	< 0.001
15–30	6151	(26.82%)	[0.35%]	7056	(26.11%)	[0.35%]		
30–40	5529	(19.29%)	[0.29%]	4623	(17.11%)	[0.30%]		
40–50	7389	(21.51%)	[0.29%]	6699	(21.41%)	[0.31%]		
50–60	6109	(15.50%)	[0.24%]	5744	(15.88%)	[0.26%]		
> 60	6565	(16.88%)	[0.26%]	7884	(19.49%)	[0.28%]		
Gender							23.78	< 0.001
female	16,320	(49.11%)	[0.36%]	15,842	(49.53%)	[0.38%]		
male	15,423	(50.89%)	[0.36%]	16,174	(50.47%)	[0.38%]		
Region							60.30	< 0.001
western	8846	(27.35%)	[0.34%]	9413	(26.45%)	[0.34%]		
central	7509	(25.51%)	[0.31%]	8064	(25.03%)	[0.31%]		
eastern	15,388	(47.13%)	[0.36%]	14,539	(48.52%)	[0.38%]		
Residence							43.30	< 0.001
urban	14,727	(50.25%)	[0.36%]	13,906	(56.33%)	[0.36%]		
rural	17,016	(49.75%)	[0.36%]	17,846	(43.67%)	[0.36%]		
Education							18.99	< 0.001
illiteracy	9162	(24.80%)	[0.30%]	8687	(24.12%)	[0.31%]		
up to junior high school	15,937	(51.88%)	[0.36%]	15,653	(53.15%)	[0.39%]		
up to vocational school	5800	(20.34%)	[0.31%]	5205	(19.60%)	[0.32%]		
bachelor degree or above	840	(2.99%)	[0.14%]	748	(3.14%)	[0.15%]		
Marital status							149.88	< 0.001
without spouse	6322	(23.3%)	[0.33%]	7661	(27.17%)	[0.35%]		
with spouse	25,417	(76.7%)	[0.33%]	24,351	(72.83%)	[0.35%]		
Occupation							4400	< 0.001
unemployment	16,347	(49.96%)	[0.36%]	8343	(26.39%)	[0.34%]		
agricultural work	7490	(21.91%)	[0.29%]	10,473	(26.98%)	[0.31%]		
self-employed	1864	(7.08%)	[0.20%]	3106	(10.83%)	[0.24%]		
employees	6042	(21.04%)	[0.31%]	10,094	(35.8%)	[0.37%]		
Self-rated health							7.75	< 0.01
good	26,371	(85.44%)	[0.24%]	26,860	(86%)	[0.25%]		
poor	5372	(14.56%)	[0.24%]	5156	(14%)	[0.25%]		

^a^Weighted proportions accounting for sampling design were used to calculate proportions and standard errors

*Person Chi2 test when comparing categories of characteristics between year 2010 and 2014

**Table 2:** Income level in year 2010 and 2014

Income	2010			2014			t	*P*
	Median	Lower quartile	Upper quartile	Median	Lower quartile	Upper quartile		
The poorest	1774	1038.2	2497.3	1500	700	2500	−10.12	< 0.001
Lower 20%	3849	3098.2	5000	5185.7	4085	6740	27.71	< 0.001
Medium 20%	5750	4730.8	7425	9366.7	7500	12,040	43.43	< 0.001
Higher 20%	8765.2	7210.5	11,200	13,450	11,001.9	17,500	39.81	< 0.001
The richest	17,500	12,806.9	25,605	24,237.5	18,712.7	33,380	6.99	< 0.001
Total	6381.3	3509	11,368.3	9836	4548	16,878.3	20.93	< 0.001

Note: Weighted proportions accounting for sampling design were used to calculate Income (CNY)

Since weighted income are skewed distribution, medians and interquartile ranges are given rather than means with standard errors

T-test *P* value for comparing income means of each categories between year 2010 and year 2013

The five income levels in 2010 and 2014 are displayed in Table 2. People reported greater income in 2014 than in 2010 (*P* < 0.001), with statistically significantly different median incomes of 9836 and 6381.3, respectively. All groups except for the poorest group had a higher median income in 2014. The poorest group earned a median income of 1500 in 2014, which is less than that reported in 2010 of 1774. As a whole, economic conditions improved over time, but the poorest in 2014 seemed to be more disadvantaged, implying the possible widening of the income gap between poor and rich.

### Concentration curve

**Fig. 1:**
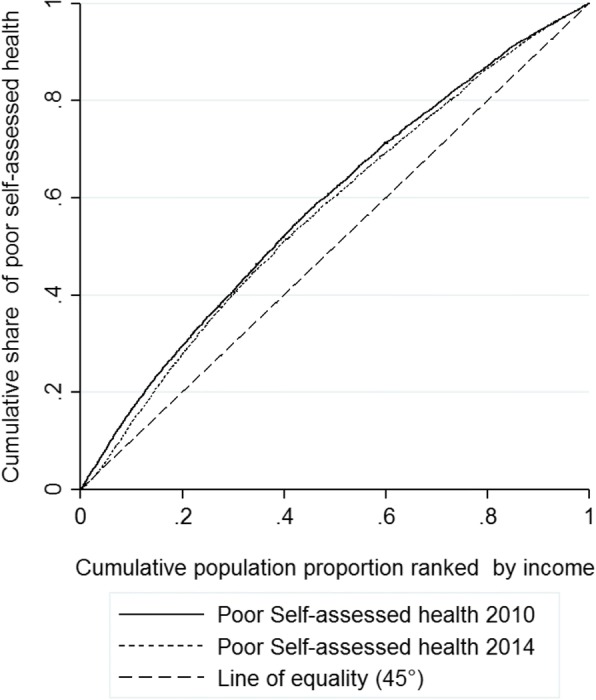
Concentration Cure of Poor Self-assessed Health and Income

The concentration curve of self-assessed health and income is shown in Fig. 1. Both self-assessed health lines were above the line of equality, with the 2014 line being closer to the line of equity. Given that the y-axis reflects poor self-assessed health, the curves show that poor health status was concentrated among the poor in both years. Comparing the curves for each year, the 2010 health line dominated the 2014 curve, with the exception of some overlap in the richest quintile, and this demonstrated that income-related health inequity decreased from 2010 to 2014. For the poorest groups, the share of people self-reporting poor health declined significantly in 2014 (change: -1.98; X^2^ = 12.74, *P* < 0.001). This implies that poor self-assessed health was more unequally distributed to the poor in 2010 than in 2014, and inequalities in self-assessed health were reduced in 2014.

### Concentration index

Table 3 shows the prevalence of poor self-assessed health in each quintile of income, the uncorrected concentration index and its standardized value after controlling for demographic covariates (see Appendix). In both years, the proportion of people reporting poor health gradually decreased with increasing income level. When comparing 2010 to 2014, a significant change was found in the poorest quintile (*P* < 0.001) in that fewer people from the poorest group reported poor health. Similar to the findings of the concentration curves, the negative values of the CIN indicated that poor health status was concentrated among the poor in both years, but inequality decreased over time, as the CIN in 2014 (uncorrected: − 0.157) was closer to zero than the CIN in 2010 (uncorrected: − 0.167). When controlling for other demographic covariates, the CIN value in 2014 (standardized: − 0.065) was closer to zero (the line of equality) than that in 2010. This provides evidence that income-related self-health inequity decreased over time. Furthermore, the standardized CIN showed greater changes across years (0.013 for standardized CIN and 0.010 for uncorrected CIN), which also demonstrated decreasing income-related inequity.

**Table 3:** Rates of poor self-assessed health in different income groups

Year	Prevalence of poor self-rated health by income quintile					Average poor self-rated health	Concentration index(SE)	Standardized CIN(SE)^a^
	Poorest	2nd	Middle	4th	Richest			
2010	22.28%	16.43%	13.71%	11.83%	9.82%	14.56%	−0.167 (0.008)	−0.088 (0.007)
2014	20.30%	16.43%	12.81%	12.43%	8.78%	14.00%	−0.157 (0.008)	−0.065 (0.007)
change	−1.98%	−0.00%	−0.90%	0.60%	−1.04%	−0.56%	0.010	0.013
Χ^2^	12.74***	0.24	5.98*	1.12	0.037	7.75**		
*P*	< 0.001	0.623	0.014	0.290	0.848	0.005		

Note: weighted proportions accounting for sampling design were used

*P < 0.05, **P < 0.01, ***P < 0.001; CIN means the concentration index

^a^controlling for age, gender, marital status, occupation, education, residence and region

### Logistic regression results

Logistic regression is the ratio scale reflecting the change between poor and rich in health status [15, 31].

Socioeconomic status ranked by income was significantly associated with poor self-assessed health in both years (Table 4). In 2010, there was a pronounced trend of those in poorer income groups reporting poor self-assessed health. When other demographic covariates were controlled for, the richest quintile was less likely to have poor self-assessed health (OR = 0.555, 95% CI: 0.484–0.636). In 2014, the 2nd quintile was not significantly different from the poorest (X^2^ = 0.55, *P* = 0.459), and the 4th quintile was not significantly different from the 3rd quintile (X^2^ = 0.41, *P* = 0.522). For the richest quintile, poor self-assessed health incurred by income remained statistically significant (OR = 0.598, 95% CI: 0.513–0696). However, there were large changes compared to the health status of the poorest quintile in 2010. The probability of reporting poor health by the richest compared to the poorest increased from 0.555 (95% CI: 0.484–0.636) to 0.598 (95% CI: 0.513–0.696). This implied that poor people had more chance to report the same self-assessed health status with the rich in 2014.

**Table 4:** Odds ratios for logistic regression of self-assessed health with socioeconomic status variables

Variables	Year 2010				Year 2014			
	OR	95% CI		*P*	OR	95% CI		*P*
Income (ref. the poorest)								
Lower 20%	0.847	0.753	0.953	0.006	0.953	0.838	1.083	0.459
Medium 20%	0.734	0.649	0.830	< 0.001	0.838	0.731	0.961	0.012
Higher 20%	0.630	0.554	0.716	< 0.001	0.799	0.697	0.916	0.001
The richest	0.555	0.484	0.636	< 0.001	0.598	0.513	0.696	< 0.001
Age (ref. 15–30)								
30–40	2.982	2.377	3.742	< 0.001	3.538	2.624	4.771	< 0.001
40–50	5.791	4.667	7.185	< 0.001	6.485	4.959	8.480	< 0.001
50–60	7.883	6.358	9.774	< 0.001	10.782	8.283	14.033	< 0.001
> 60	10.116	8.195	12.487	< 0.001	11.968	9.320	15.368	< 0.001
Gender (ref. female)								
Male	0.813	0.746	0.886	< 0.001	0.821	0.746	0.904	< 0.001
Marital status (ref. single)								
Married	0.936	0.825	1.062	0.307	0.985	0.857	1.131	0.826
Occupation (ref. unemployment)								
Agricultural	0.805	0.729	0.889	< 0.001	0.655	0.580	0.740	< 0.001
Self-employed	0.517	0.413	0.648	< 0.001	0.509	0.416	0.622	< 0.001
employees	0.558	0.477	0.654	< 0.001	0.544	0.464	0.637	< 0.001
Education (ref. illiteracy)								
up to junior high school	0.671	0.609	0.739	< 0.001	0.568	0.511	0.632	< 0.001
up to vocational school	0.577	0.496	0.672	< 0.001	0.423	0.355	0.504	< 0.001
bachelor degree or above	0.543	0.341	0.865	0.010	0.342	0.202	0.578	< 0.001
Residence (ref. rural)								
urban	0.820	0.749	0.899	< 0.001	0.839	0.759	0.927	< 0.01
Region (ref. west)								
Central	1.010	0.903	1.129	0.868	0.892	0.789	1.008	0.068
East	0.961	0.865	1.067	0.455	0.931	0.831	1.043	0.217
Constant	0.098	0.079	0.122	< 0.001	0.090	0.070	0.116	< 0.01
	R^2^ = 0.136				R^2^ = 0.154			

Note: weighted proportions accounting for sampling design were used. Models in both years controlled for age, gender, marital status, occupation, education, residence and region

## Discussion

This work analysed the latest nationally representative data from CFPS 2010 and 2014 using the standardized concentration index after controlling for demographic variables, urban-rural differences and district differences. The logistic regression generated the same results as the standardized CIN, which shows the robustness of the findings. This study measured the changes in self-assessed health inequity among different income groups among adults age 15 years and older from 2010 to 2014 in China. The decrease in the concentration curve slope implies that the health distribution among income groups moved close to the line of equality, with the proportion of people reporting poor health dropping from 22.28 to 20.30% (*P* < 0.001) in the poorest group, which indicates that the reduction in health inequity can mainly be attributed to improvements in health among the poor. In 2010, the proportion of respondents self-reporting poor health gradually decreased with increasing income. However, the poorest and lower 20% were not significantly different in 2014, and neither were the medium and higher 20%. The Odds Ratio between the poorest and richest in poor self-assessed health status increase from 0.555 to 0.598, indicating that moving from the poor to the rich decrease poor SAH by 0.598 unit in 2014, less than 0.555 in 2010. This finding is observed because self-assessed health among the poorest improved, and the health gap between the poor and rich thus narrowed.

Self-assessed health is not only a measure of health status but also a strong predictor of morbidity, mortality, mental health and chronic diseases. There are many recent studies of the relationship between self-assessed health and socio-economic determinants in China. Sun et al. (2009) examined the interaction between poverty and self-assessed health in urban China and showed that lack of social capital was a good predictor of poor self-assessed health [33]. Wei and Kanavos found the poor were less likely to report their health status as “excellent or good” and this income-related inequality was more pronounced for urban population [30]. Zhong et al. (2013) reported the inverse relationship between income level and poor self-assessed health using data from the China Health and Retirement Longitudinal Study in 2011, which is consistent with our findings from the CFPS 2010 [34]. Cheng et al. (2014) claimed that NRCMS had significantly improved the elderly enrolless’ activities of daily living and cognitive function but failed to better SAH, using panel data from 2005 to 2008 waves of Chinese Longitudinal Healthy Longevity Survey [35]. In the contrary, our study showed a positive achievement in improving SAH nationwide from 2010 to 2014. Guo and Shi (2015) concluded that socio-economic inequalities in self-assessed health of the elderly was less pronounced than the health inequity related to differences in education [36]. Liu et al. (2016) found income-related self-assessed health inequities among rural Chinese citizens with a concentration index of − 0.088 [37]. Studies of self-assessed health and health equity are becoming more and more prevalent.

Consistent with our results, many studies have identified income-related health inequities and found that poverty is strongly associated with poor self-assessed health [30, 34–38]. However, most prior studies used cross-sectional data on subpopulations, few examined changes over time, and even the effect of NRCMS in improving SAH for the elderly was denied in the early stage from 2005 to 2008. Our study did not talk about a brand-new topic, but provided the latest evidence on the effect of health reform at 2009 in the perspective of longitudinal changes. This study identified a reduction in health inequity from 2010 to 2014. This implies that the three-fold policies implemented by the government, consisting of the equalization of essential public health services, the expansion of health insurance coverage and the strengthening of the capacity of primary health care, reduced barriers to accessing and using effective health care and prevention services by socially disadvantaged and vulnerable groups without incurring financial burdens.

Since the WHO called for the closing of the health equity gap through action on the social determinants of health [3, 39, 40], countries and governments have incorporated equity goals into national public programmes. Primary health care can be used to pursue social justice, and the right to better health for all can help the health system respond better and faster to people’s health needs. One key element to achieve this goal is reducing exclusion and social disparities in health through universal coverage reforms [41, 42]. However, there are shortcomings of health care delivery in the present health systems worldwide. First, the wealthiest people consume the most care, and their health care needs are often less acute than those of the poor. Second, people lacking social protection and capital will be confronted with catastrophic expenses when paying for health care out-of-pocket. Third, health providers neglect specialization of health delivery for disadvantaged subgroups. Health services for marginalized groups are under-resourced, and the safety and quality of care is hard to ensure. Hence, a WHO report outlined four sets of PHC reforms to move towards universal access and social health protection, reorganize health services as primary care, integrate public health actions with primary care and strengthen leadership in complex health systems [43]. According to a World Bank report of how 24 developing countries are implementing bottom-up universal health coverage reforms, 26 UHC programmes followed a bottom-up approach by expanding coverage and ensuring that the poor has access to health care without suffering financing hardship [44].

Without off-the-shelf solutions, China has successfully achieved expansion in coverage of affordable health access, but there is a long way to go to strengthen primary care for all. There is great room for improvement in China’s health system to reorganize health services as primary care and strengthen leadership in complex systems. In fact, the proportion of primary health care utilization is declining among total health services [45]. Cooperation between health-related sectors is dissatisfactory [46–48]. China needs to address these weaknesses in the near future. This necessity has been clearly put forward in “Health in All Policies” in the Plan of Health China 2030 [49], and whether the goal can be reached remains to be seen and evaluated.

The findings of this study will not only provide evidence to improve health policy in China but will also provide a reference for other low- and middle-income countries (LMICs) that are attempting to improve health equity. It is worth noting that China as a developing country with the fastest economic growth, some policies may not be suitable in the context of other developing countries. The rapid economy growth and increasing health input in China have ensured that the financial investment can continue to strengthen primary health capacity and raise funding targeted for vulnerable groups. The intensity and scale of strengthening primary health care in other developing countries may be limited, but this is both a powerful tool recommended by international organizations and has also been verified by Chinese practice. Under the circumstances of huge health disparities, it is still an effective way to improve health equity by promoting capacity of primary health care in health system and ensuring access to health services for everyone. However, there are also several issues that need to be addressed in the future work: First, health funding through fiscal revenue is restricted. The government should consider multiple financing sources and mobilize social capital to ensure sustainable development. Second, there may be problems of inefficiency in funds allocation. Unifying subsidy standard and same services are not equal to fairness. In addition, it should pay attention to the health needs of different populations and provide people-oriented individual services instead of simply package services across all groups. Third, government should strictly monitor the delivery of health services and ensure quality and suitable care for everyone.

Our study has several limitations. First, the time interval is short (five years) and the panel data is not available. It is concerned that change of people’s health condition within 5 years may not obvious enough to be reflected by significant variance in the indicators, which may also underestimate the impact of health interventions. In particular, good or sub-health people without chronic or severe diseases may not feel minor changes in health conditions. On the other hand, because of cost restriction and difficulty in implementation, this survey is not strictly a longitudinal tracking survey with panel data, and therefore cannot control the interference of certain time-varying factors. Second, absence of objective indicators. Disease diagnosis information was affected by the interviewer’s recalling bias and some even refused for privacy concerns, which generated many missing values. In addition, only a Five-point Likert Scale measurement was used to report SAH, rather than a standardized instrument of EQ5D. Therefore, detailed health information of SAH at each dimension is lacking, and it is hard to further explore the path of health policy on improving SAH. Subsequent studies should focus on the components of SAH to provide a key point for further promoting health policies. Third, the controversy of SAH representing health status. Since SAH is closely related to subjective perception and its standard varies greatly among those of different cultural background and religious belief, people with the same health condition may report different SAH. Moreover, it is argued that econometric analysis of SAH showed a near-zero effect [15], consistent with our findings in regression models (low R square). A deeper understanding of SAH and its components is important before jumping to the conclusion. Considering the above factors and the previous relevant studies in China, we finally selected the practicable but not ideal indicator-SAH-as general health status.

## Conclusions

With a series of health reforms dedicated to increasing access to care for the poor, the self-assessed health gap between income groups in China decreased from 2010 to 2014. This change is related to government health policy efforts to expand health insurance coverage and increase access to primary health care. China’s endeavours to reduce health inequity are in accordance with the WHO recommendation and actions in other developing countries. However, health differences remain. To improve health equity, China should promote the implementation of “Health in All Policies” through a more effective primary care system and closer cooperation between health departments.

## Appendix

**Table 5:** Regression results for demographic standardization of the concentration index

Variables	Year 2010				Year 2014			
	Coefficient	95% CI		*P*	Coefficient	95% CI		*P*
Income	−0.088^a^	−0.102	−0.074	< 0.001	−0.065^a^	−0.079	−0.051	< 0.001
Age (ref. 15–30))								
30–40	0.060	0.047	0.073	< 0.001	0.056	0.043	0.070	< 0.001
40–50	0.127	0.115	0.140	< 0.001	0.106	0.093	0.120	< 0.001
50–60	0.179	0.165	0.193	< 0.001	0.180	0.166	0.195	< 0.001
> 60	0.245	0.231	0.259	< 0.001	0.227	0.213	0.242	< 0.001
Gender (ref. female)								
Male	−0.026	−0.034	−0.018	< 0.001	− 0.026	− 0.034	− 0.018	< 0.001
Education (ref. illiteracy)								
up to junior high school	−0.077	− 0.088	− 0.066	< 0.001	− 0.105	−0.115	− 0.094	< 0.001
up to vocational school	−0.084	− 0.099	− 0.070	< 0.001	− 0.120	−0.134	− 0.106	< 0.001
bachelor degree or above	−0.070	− 0.095	−0.044	< 0.001	− 0.115	− 0.140	−0.090	< 0.001
Marital status (ref. single)								
Married	−0.012	−0.023	− 0.001	0.028	− 0.005	−0.016	0.006	0.397
Occupation (ref. unemployment)								
Agricultural	−0.028	−0.039	− 0.018	< 0.001	− 0.049	−0.061	− 0.038	< 0.001
Self-employed	−0.049	− 0.064	− 0.033	< 0.001	− 0.066	−0.080	− 0.051	< 0.001
employees	−0.038	− 0.049	− 0.027	< 0.001	− 0.050	−0.061	− 0.039	< 0.001
Residence (ref. rural)								
urban	−0.024	−0.033	− 0.016	< 0.001	− 0.022	−0.030	− 0.013	< 0.001
Region (ref. west)								
Central	0.002	−0.009	0.013	0.719	−0.010	−0.020	0.001	0.079
East	−0.003	−0.012	0.007	0.579	−0.002	−0.011	0.008	0.068
Constant	0.201	0.186	0.216	< 0.001	0.225	0.209	0.241	< 0.001
	R^2^ = 0.108				R^2^ = 0.121			

Note: ^a^coefficient for income is the standardized concentration index

Models in both year are controlled for age, gender, education, marital status, occupation, residence and region

## Abbreviations

CFPS: China Family Panel Studies
CIN: Concentration index
LMICs: Low- and middle-income countries
